# LRR‐UNet: A Deep Unfolding Network With Low‐Rank Recovery for EEG Signal Denoising

**DOI:** 10.1111/cns.70632

**Published:** 2025-10-27

**Authors:** Xiaoxiong Yue, Liangfu Lu, Haipeng Liu, Yunliang Zang

**Affiliations:** ^1^ Academy of Medical Engineering, and Translational Medicine Tianjin University Tianjin China; ^2^ Centre for Intelligent Healthcare Coventry University Coventry UK; ^3^ Xiamen Intretech Inc Xiamen Fujian China

**Keywords:** deep unfolding network, denoising, EEG signals, low‐rank recovery, U‐Net

## Abstract

**Background:**

Electroencephalogram (EEG) signals are crucial for brain–computer interface research but are highly susceptible to noise contamination, necessitating effective denoising. While deep learning has been widely applied, its “black‐box” nature limits interpretability. In contrast, traditional model‐based methods like Low‐Rank Recovery (LRR) offer strong interpretability by decomposing signals into low‐rank and sparse components.

**Objective:**

This paper aims to develop an interpretable deep‐learning model for EEG denoising that combines the performance of deep learning with the interpretability of traditional LRR methods.

**Methods:**

We propose LRR‐Unet, a deep unfolding network that transforms the traditional iterative LRR algorithm into a neural network architecture. Specifically, the time‐consuming Singular Value Decomposition (SVD) and sparse optimization processes in LRR are replaced with learnable neural network modules.

**Results:**

Extensive experiments demonstrate that LRR‐Unet outperforms other state‐of‐the‐art models in removing ocular and electromyographic artifacts, achieving superior performance on both quantitative and qualitative metrics. Furthermore, in downstream classification tasks, EEG signals preprocessed with LRR‐Unet yield better results across various evaluation indicators.

**Conclusion:**

The proposed LRR‐Unet provides an effective and interpretable solution for EEG denoising. Its superiority in denoising performance and practical utility in enhancing downstream application performance is validated through comprehensive experiments.

## Graphical abstract

Drawing on the intrinsic properties of EEG signals and noise, we developed an EEG denoising algorithm that integrates low‐rank recovery theory with deep learning. Supported by a physical model, this algorithm demonstrates superior denoising performance on relevant datasets.

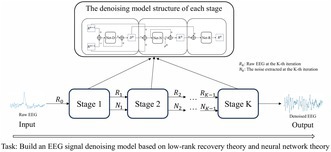

## 1 Introduction

Common artifacts in EEG signals include ocular artifacts (EOG), electromyographic artifacts (EMG), and powerline interference (50/60 Hz). Among these, powerline interference exhibits fixed and singular frequency characteristics, which can be effectively removed through hardware filtering (e.g., notch filters) or digital filtering (e.g., band‐stop filters). However, the low‐frequency components of EOG artifacts significantly overlap with EEG alpha waves (8–13 Hz) and theta waves (4–8 Hz), directly interfering with resting‐state EEG analysis (e.g., alpha wave power spectrum calculation). Similarly, the high‐frequency components of EMG artifacts may obscure EEG gamma waves (30–100 Hz), which are closely associated with higher cognitive functions such as information processing and attention. Such contamination can substantially compromise feature extraction in task‐related EEG studies. Therefore, the removal of electrooculogram (EOG) and electromyographic (EMG) artifacts becomes particularly crucial. These two types of artifacts severely distort EEG signals, significantly degrading signal quality and analytical accuracy [1]. EOG artifacts, primarily generated by blinks and eye movements, produce large‐amplitude potential variations on the scalp that can easily mask meaningful neural information in EEG signals. This interference is especially problematic in event‐related potential (ERP) analysis, where EOG contamination may lead researchers to misinterpret artifacts as genuine neural responses, potentially yielding erroneous conclusions. EMG artifacts, arising from facial, neck, or other muscle activities, exhibit substantial spectral overlap with EEG signals, making them particularly challenging to separate during signal processing [2]. With the continuous advancement of brain–computer interface (BCI) technology, EEG signal quality has become a critical determinant of BCI system performance. Consequently, effective removal of EOG and EMG artifacts is essential for enhancing the stability and accuracy of BCI systems.

There has been extensive research on EEG signal denoising algorithms, which can be mainly divided into traditional algorithms and deep learning algorithms. Traditional algorithms include wavelet transform, empirical mode decomposition (EMD), independent component analysis (ICA), etc. However, these traditional algorithms also have some drawbacks. For wavelet transform, researchers are troubled by the loss of effective signals during EEG signal denoising and the distortion of signals caused by threshold denoising [3]. Complete ensemble empirical mode decomposition (CEEMD) is an improvement on EMD that alleviates the problem of mode mixing by adding two pairs of opposite white noise. However, selecting effective modes in CEEMD decomposition is challenging, and using only local reconstruction methods for signal‐dominant modes will inevitably result in the loss of useful information [4]. ICA has the advantages of not requiring prior knowledge and automatically separating source signals in EEG signal denoising, effectively removing noise such as EOG and EMG. However, this algorithm has high computational complexity, requires a large amount of data, and is limited in effectiveness when source signals are highly correlated, requiring manual selection of independent components [5].

Due to the increase in computing power and the publication of numerous EEG signal datasets, deep learning‐based EEG signal denoising algorithms have developed rapidly. Deep learning algorithms used for EEG signal denoising mainly include feedforward neural networks (FNN), convolutional neural networks (CNN), and recurrent neural networks (RNN). However, these algorithms are considered black‐box models that, while achieving satisfactory denoising results, lack interpretability in the denoising process [6].

In recent years, researchers have considered the strengths and weaknesses of traditional models and deep learning models, developing a new algorithm that combines the advantages of both: the deep unfolding network. This network aims to bridge the gap between iterative algorithms and neural networks. This technique has been applied in multiple fields and has attracted widespread attention, as described in Refs. [7, 8, 9]. The core of the deep unfolding network lies in unfolding the iterative solution algorithm of an existing model at the iterative level, with each iteration performed by a neural network to form the network structure. Subsequently, the hyperparameters of this network are updated through backpropagation of errors. By establishing a systematic and precise connection between traditional iterative algorithms and neural networks, deep unfolding networks demonstrate great potential in creating interpretable networks [10].

In the field of EEG signal denoising, research based on low‐rank representation (LRR) theory remains relatively limited. However, similar to LRR‐based image denoising, the EEG denoising process can also be conceptualized as the estimation of low‐rank clean signals and the extraction of sparse noise components. This process is primarily implemented through singular value decomposition (SVD) and soft thresholding of the signals. Nevertheless, when employing neural networks to address this problem, it necessitates performing SVD processing on each neural tensor during every forward propagation, which presents significant challenges in terms of computational time and accuracy. Regarding one‐dimensional EEG signals, they inherently lack low‐rank properties since their rank is essentially 1. However, EEG signals exhibit temporal correlation characteristics—that is, across different time windows, the brain's fundamental functions and activity patterns may remain consistent or demonstrate certain regularities [11]. Consequently, signals from different time windows of one‐dimensional EEG data in the time domain share certain similarities, and this similarity can be considered a form of low‐rank property. Notably, EMG noise generated by muscle contractions manifests as high‐frequency, short‐pulse interference, while EOG noise caused by eye movements exhibits low‐frequency, high‐amplitude, and nonperiodic characteristics. Both types belong to sparse noise (with energy concentrated at specific time points or frequency bands), forming a sharp contrast with the low‐rank structure of EEG signals. Therefore, while previous deep learning models primarily relied on data‐driven approaches, the model proposed in this study fully incorporates prior knowledge about both EEG signals and their associated noise characteristics.

Therefore, based on LRR theory and combined with the deep unfolding algorithm, we have designed a new EEG signal denoising network called LRR‐Unet. For the extraction of clean EEG signals, we have designed a Net‐D to approximate this process. This network adopts a network structure similar to Unet, enabling it to learn features in EEG signals from different receptive fields and avoiding operations such as SVD. For noise extraction, we use a convolutional neural network Net‐N for approximation, thereby avoiding the problem of sparse optimization. Finally, a Net‐R is designed to combine the denoised EEG signal and noise to obtain a new original signal for the next iteration. Specifically, the contributions of this paper are as follows:

1. We have designed an EEG signal denoising network that combines the interpretability of traditional models with the powerful learning capabilities of deep learning.
2. The designed Net‐D and Net‐N can effectively extract clean EEG signals and noise from the original EEG signal, avoiding the problems of SVD and sparse optimization. A Net‐R is finally designed to combine the denoised EEG signal and noise to obtain a new original signal for the next iteration.
3. On open‐source EEG signal datasets such as EEGDenoiseNet, the denoising performance of this algorithm surpasses other advanced algorithms, proving the feasibility of the model.

## 2 Related Work

### 2.1 Traditional Denoising Algorithm

Wavelet transform possesses excellent time‐frequency characteristics, making it well‐suited for processing nonlinear and nonstationary EEG signals. By selecting an appropriate wavelet function, the EEG signal is decomposed into wavelet coefficients at different frequency layers. Thresholding is then applied to remove wavelet coefficients corresponding to noise components, and the remaining wavelet coefficients are reconstructed to recover the denoised EEG signal. The wavelet approach effectively reduces noise while preserving important signal features [12]. Similarly, EMD decomposes EEG signals into multiple intrinsic mode functions (IMFs); after filtering out noise‐related IMFs, the reconstruction of remaining components yields denoised signals through this adaptive decomposition framework [13]. Independent component analysis (ICA) first analyzes multiple observed signals to extract independent components, then identifies and separates noise‐related independent components, and finally reconstructs the noise‐free EEG signal by setting noise components to zero or modifying the mixing matrix. This process effectively suppresses noise, improving the signal‐to‐noise ratio and clarity of the signal [14].

However, these traditional algorithms also have several drawbacks. In wavelet thresholding denoising, the selection of the wavelet basis and the number of decomposition levels is subjective, and improper choices may lead to the loss of valid information and signal distortion [3]. While EMD suffers from mode mixing, its improved variant CEEMD addresses this by adding counter‐phase white noise pairs. Nevertheless, CEEMD still faces challenges in optimal mode selection [4]. ICA struggles to determine the exact number of independent sources, particularly in limited‐electrode EEG recordings, potentially causing valid neural components to be discarded with noise. Additionally, for random, short‐duration noise, ICA may not effectively separate it, and noise in long‐duration signals may be difficult to fully identify [5].

### 2.2 Denoising Algorithms Based on Deep Learning

In recent years, deep learning has been widely used in computer vision [15, 16, 17, 18] and natural language processing [19, 20, 21], achieving remarkable success. For EEG signal denoising, deep learning has also demonstrated significant effectiveness.

Sun et al. [22] proposed a one‐dimensional residual Convolutional Neural Network (1D‐ResCNN) model based on CNN architecture, which achieved satisfactory denoising performance. Yu et al. [23] developed an end‐to‐end deep learning framework named DeepSeparator, where the encoder captures and amplifies features in raw EEG signals, the decomposer extracts trends while detecting and suppressing artifacts in the embedding space, and the decoder reconstructs both the clean EEG signals and artifacts. Pu et al. [24] introduced a Transformer architecture that captures nonlocal self‐similarity in EEG signals through self‐attention mechanisms, while simultaneously utilizing local self‐similarity in feedforward blocks, thereby significantly mitigating the negative impact of noise and outliers on EEG signals. Fengjie Wu et al. [25] proposed an EEG denoising model for discriminatively removing long‐ and short‐term distributed artifacts: the short‐term network captures local variations to remove short‐term artifacts, while the long‐term network reconstructs signals affected by long‐term artifacts to improve denoising quality. Pu Zeng et al. [26] presented a Task‐Oriented EEG Denoising Generative Adversarial Network (TOED‐GAN) that employs a generator to decompose raw EEG signals and reconstruct clean signals, while the discriminator learns to distinguish between generated signals and real clean signals, thereby achieving effective denoising. Chuang C H [27] and others have constructed the IC‐U‐Net for EEG denoising. This algorithm is based on the U‐Net architecture and a series of loss functions, using synthetically mixed brain and nonbrain independent components (ICs) separated by ICA and ICLabel. During the training process, it identifies model parameters that minimize the difference between the mixed brain ICs and the outputs of the model.

However, these algorithms are considered black‐box models. Although they can achieve satisfactory denoising results, they lack interpretability in the denoising process. Deep unfolding networks (DUNs) serve as a bridge connecting traditional models and deep learning algorithms [28]. Among them, deep unfolding networks based on the LRR algorithm have been widely used in image denoising and other fields. Since image denoising and EEG signal denoising are essentially the same in nature, research on deep unfolding networks based on the LRR algorithm for EEG signal denoising holds significant importance.

## 3 LRR‐Unet Model

### 3.1 Problem Formulation

For a segment of brain electrical signal *R* that is contaminated by noise, it can be regarded as the sum of a clean brain electrical signal *D* and a noise signal *N*:

(1) R=D+N

where $R,D,N\in R^{n}$ (*n* represents the number of sampling points in the EEG signal), in general low‐rank representation (LRR) theory, we attempt to recover the low‐rank clean brain electrical signal *D* and the sparse noise signal *N* from the observed signal *R*. Typically, the following objective function is used [29]:

(2) $$\begin{array}{c} \min_{D.N}\;\mathrm{rank}\left(D\right)+\mu {\left\|N\right\|}_{0}\;\mathrm{st}.R=D+N \end{array}$$

where μ is a positive balancing parameter, ${\left\|\cdot \right\|}_{0}$ denotes the $l_{0}$ norm, which represents the number of nonzero elements in the noise signal N. The minimization problems for the $l_{0}$ norm and the rank function are NP‐Hard. Based on the ideas of Chandrasekaran [30] and Fazel et al., $L_{1}$ minimization is the tightest convex relaxation of $l_{0}$ minimization, and the nuclear norm is the tightest convex relaxation of the rank function. Therefore, $l_{1}$ norm minimization is used as an approximation to solve the $l_{0}$ norm minimization problem, and the nuclear norm is used as an approximation for the rank function:

(3) $$\begin{array}{c} \min_{D,N}{\left\|D\right\|}_{*}+\mu {\left\|N\right\|}_{1}\;\mathrm{st}.R=D+N \end{array}$$

In the equation, ${\left\|D\right\|}_{*}$ represents the sum of the singular values obtained after performing SVD on *D*, and ${\left\|N\right\|}_{1}$ represents the sum of the absolute values of the elements in *N*. However, for one‐dimensional EEG signals, the nuclear norm in the above equation finds it difficult to capture the underlying low‐rank properties. At the same time, for complex noise signals, the $l_{1}$ norm also struggles to capture the actual constraints involved. Therefore, we use $U\left(D\right)$ and $S\left(N\right)$ to incorporate prior knowledge about the clean EEG signal and noise signal, respectively, in the noisy EEG signal:

(4) $$\begin{array}{c} \min_{D,N}U\left(D\right)+\mathrm{\mu S}\left(N\right)\;\mathrm{st}.R=D+N \end{array}$$

Meanwhile, to simplify the computational complexity introduced by Lagrange multipliers, we directly use the simpler and more intuitive $l_{2}$ norm to convert the above problem into an unconstrained optimization problem:

(5) $$\begin{array}{c} \min_{D,N}U\left(D\right)+\mathrm{\mu S}\left(N\right)+\frac{\beta }{2}{\left\|R-D-N\right\|}_{F}^{2} \end{array}$$

where β is a penalty coefficient, and ${\left\|\cdot \right\|}_{F}$ represents the Frobenius norm (F‐norm). Based on the above formula, we can separately optimize and solve for the clean EEG signal and the noise signal within an iterative framework.

### 3.2 Solving Model

Updating D: This process is the extraction of the clean EEG signal. This problem can be expressed using the following formula:

(6) $$\begin{array}{c} D={\text{argmin}}_{D}U\left(D\right)+\frac{\beta }{2}{\left\|R-D-N\right\|}_{F}^{2} \end{array}$$

Here, ${\text{argmin}}_{D}$ represents the value of D that minimizes the given formula, $U\left(D\right)$ denotes a constraint imposed on D in this study, and β is a penalty coefficient. According to traditional LRR algorithms, the solution to the above equation is achieved by approximating $U\left(D\right)$ as ${\left\|D\right\|}_{*}$, and then minimizing the sum of the nuclear norm and the $l_{2}$ norm. This problem can be expressed using the following formula:

(7) $$\begin{array}{c} D={\mathrm{SVT}}_{\alpha }\left(R-N\right) \end{array}$$

where ${\mathrm{SVT}}_{\alpha }\left(\cdot \right)$ represents performing SVD on the elements and then applying singular value shrinkage with a threshold of α [30]. However, in deep unfolding networks, we approximate the solution to the above formula by constructing a neural network. This means that we need to perform SVD operations during each forward propagation, which poses significant challenges in terms of accuracy and computational time [31]. Additionally, for one‐dimensional EEG signals, the nuclear norm finds it difficult to capture the underlying low‐rank properties. Therefore, in this study, we did not adopt the traditional SVD decomposition for solution, but instead designed an approximate operator to extract the clean EEG signal:

(8) $$\begin{array}{c} D=\text{appro}\left(R-N\right) \end{array}$$

EEG signals are temporally correlated, and the basic activity patterns of the brain exhibit certain regularities across different time windows [11]. Therefore, one‐dimensional EEG signals share certain similarities within different time windows in the time domain. Unet [32] is a deep learning architecture suitable for one‐dimensional data (such as time series). It captures multi‐level features of the data during the encoding (down sampling) process (which is conducive to learning the low‐rank properties in various time windows) and fuses low‐level and high‐level features through skip connections during the decoding (up sampling) process, which helps to recover detailed information. Therefore, this paper designs a Unet to approximate the $\text{appro}\left(\cdot \right)$ operator, leveraging the powerful nonlinear capabilities of neural networks to extract deep features from EEG signals in a data‐driven manner, which will be described in detail in the following chapters.

Updating N: Similar to the updating of D, the extraction of noise signals can be expressed using the following formula:

(9) $$\begin{array}{c} N={\text{argmin}}_{N}\mathrm{\mu S}\left(N\right)+\frac{\beta }{2}{\left\|R-D-N\right\|}_{F}^{2} \end{array}$$

where μ is a positive balancing parameter, In the previous sections, it was discussed that a commonly used optimization method for the above equation is to add an $L_{1}$ norm constraint to the noise and then solve it using soft thresholding. However, performing soft thresholding operations in neural networks is also challenging [33]. Meanwhile, the complex noise signals in EEG signals are difficult to capture using a single sparsity constraint. Therefore, to address the above issues, we consider the Taylor expansion of $S\left(N\right)$. For a differentiable function $f\left(x\right)$, $\nabla f\left(x\right)$ is Lipschitz continuous (i.e., $\forall x_{1},x_{2}:\left\|\nabla f\left(x_{1}\right)-\nabla f\left(x_{2}\right)\right\|\leq l\left\|x_{1}-x_{2}\right\|$), where *l* is a constant [34]. In this case, $f\left(x\right)$ can be approximated using a Taylor series expansion at a fixed point $x_{0}$:

(10) $$\begin{array}{c} f\left(x\right)\leq \hat{f}\left(x,x_{0}\right)=\frac{l}{2}{\left\|x-x_{0}+\frac{1}{l}f\left(x_{0}\right)\right\|}_{2}^{2}+C \end{array}$$

Based on this, we can approximate $S\left(N\right)$ using the result of the previous iteration $N^{k-1}$ with the following formula:

(11) $$\begin{array}{c} \hat{S}\left(N,N^{k-1}\right)=\frac{l_{s}}{2}{\left\|N-N^{k-1}+\frac{1}{l_{s}}f\left(N^{k-1}\right)\right\|}_{2}^{2}+C_{s} \end{array}$$

where $l_{s}$ is the Lipschitz constant related to $S\left(N\right)$, and $C_{s}=-\frac{1}{2l}{\left\|\nabla S\left(N^{k-1}\right)\right\|}_{2}^{2}+S\left(N^{k-1}\right)$ is a constant. Therefore, Equation (9) can be further solved using the following formula:

(12) $$\begin{array}{l} N={\text{argmin}}_{N}\mathrm{\mu S}\left(N\right)+\frac{\beta }{2}{\left\|R-D-N\right\|}_{F}^{2}\\ =\frac{\mu l_{s}}{2}{\left\|N-N^{k-1}+\frac{1}{l_{s}}f\left(N^{k-1}\right)\right\|}_{2}^{2}\\ \;+\frac{\beta }{2}{\left\|R-D-N\right\|}_{F}^{2} \end{array}$$

By taking the derivative of the equation and setting it to zero, we can obtain a closed‐form solution for updating N at the *k*‐th step:

(13) $$\begin{array}{c} N^{k}=\frac{\mu l_{s}}{\mu l_{s}+\beta }N^{k-1}+\frac{\beta }{\mu l_{s}+\beta }\left(R^{k-1}-D^{k}\right)-\frac{\mu }{\mu l_{s}+\beta }\nabla S\left(N^{k-1}\right) \end{array}$$

Among the three coefficients, they are all constants. Let $\delta =\frac{\mu l_{s}}{\mu l_{s}+\beta }$, $\rho =\frac{\mu }{\mu l_{s}+\beta }$:

(14) $$\begin{array}{c} N^{k}=\delta N^{k-1}+\left(1-\delta \right)\left(R^{k-1}-D^{k}\right)-\rho \nabla S\left(N^{k-1}\right) \end{array}$$

### 3.3 Network Architecture

**FIGURE 1:**
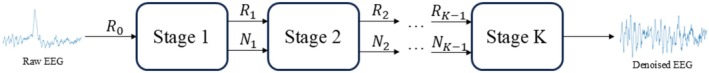
The entire denoising process of the denoising model is described. $R_{0}$ represents the original signal contaminated by noise, and stage *k* represents the k‐th iterative operation. The outputs $R_{K}$ and $N_{K}$ at each stage represent the denoised signal and the separated noise signal respectively. The output *R* of the last iteration is the final denoised signal.

In this chapter, we elaborate on the overall architecture and detailed components of the LRR‐Unet based on the optimization equation from the previous chapter. As shown in Figure 1, the input to the network is the noise‐contaminated EEG signal $Y\in R^{n}$, where n represents the number of sampling points in the EEG signal. The initial parameters are updated as $R^{0}=Y$, $N^{0}=0$, and these parameters are then fed into the network for *k* iterations, where stage *k* represents the computational process of the *k*‐th iteration. This process integrates the iterative solution of the model into the designed neural network, ultimately outputting the denoised EEG signal after *k* iterations.

**FIGURE 2:**
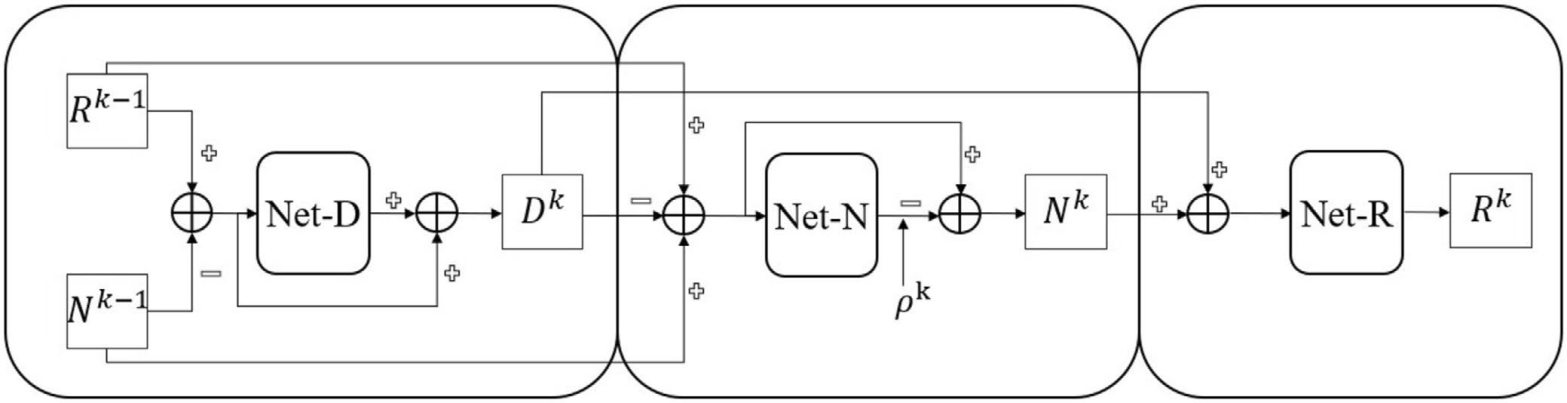
The overall structure of the denoising model proposed in this paper. Among them, the main function of Net‐D is to extract the clean electroencephalogram (EEG) signals, the main function of Net‐N is to extract the noise signals, and the main function of Net‐R is to fuse the previously extracted intermediate clean signals and noise signals to obtain the input for the next iteration.

During the (*k* − 1)‐th iteration solution, the clean EEG signal $D^{k-1}$ is extracted by Net‐D, while the noise component $N^{k-1}$ is extracted by Net‐N. Subsequently, Net‐R integrates $D^{k-1}$ with $N^{k-1}$ to generate the input $R^{k-1}$ for the next iteration. Using $R^{k-1}$ and $N^{k-1}$ as inputs, the k‐th iteration is then performed, as illustrated in Figure 2. Here, $R^{k-1}$ and $N^{k-1}$ represent the raw EEG signal and noise obtained from the (*k* − 1)‐th iteration respectively, $D^{k}$ denotes the clean EEG signal (i.e., the final denoising result) produced by the *k*‐th iteration, $\rho^{k}$ is the learnable scalar parameter for the *k*‐th iteration, and $R^{k}$ with $N^{k}$ correspond to the raw EEG signal and noise derived from the *k*‐th iteration, respectively. The detailed architectural designs of each subnetwork will be introduced in the following section.

#### 3.3.1 Denoising EEG Estimation Module (Net‐D)

In Equation (8), we designed an approximation operator $\text{appro}\left(.\right)$ to extract the clean EEG signal. Inspired by [32, 35, 36], Unet [37] is a deep learning architecture suitable for one‐dimensional data (such as time series). It captures multi‐level features of the data during the encoding (down sampling) process, which facilitates learning the low‐rank property in various time windows of the one‐dimensional EEG signal. During the decoding (up sampling) process, skip connections are used to fuse low‐level and high‐level features, contributing to the recovery of detailed information in the EEG signal. Therefore, we designed a Unet to extract the clean EEG signal and adopted an efficient residual structure to enhance the signal.

(15) $$\begin{array}{l} D^{k}=\text{appro}\left(R^{k-1}-N^{k-1}\right)\\ =R^{k-1}-N^{k-1}+\text{NetD}\left(R^{k-1}-N^{k-1}\right) \end{array}$$

**FIGURE 3:**
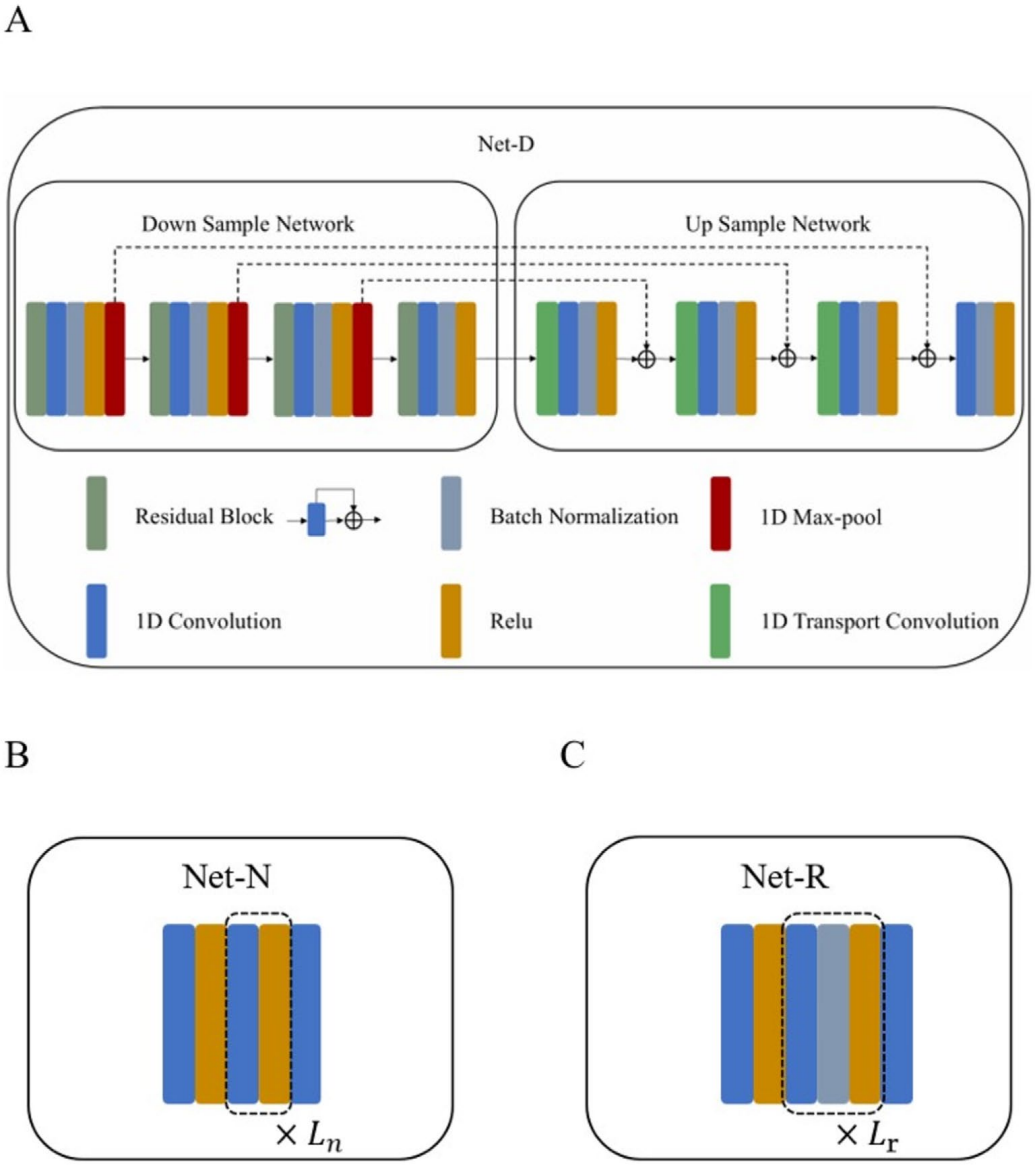
The detailed structure of each module of the denoising model proposed in this paper.

The design of Net‐D is illustrated in Figure 3A. This network is a type of Unet. In the down sampling stage, the first three convolutional layers consist of $\left[\mathrm{Res}+\text{Conv}+\mathrm{BN}+\text{ReLU}+\mathrm{MP}\right]$, where MP denotes max pooling, and the last convolutional layer is $\left[\mathrm{Res}+\text{Conv}+\mathrm{BN}+\text{ReLU}\right]$. The output of the ReLU layer in the first three convolutional layers is fused with the output of the upsampling stage using skip connections to achieve feature fusion. During the downsampling process, the scale of the input EEG signal decreases continuously, while the number of channels increases, allowing for the extraction of features at different scales [38]. This is beneficial for capturing the hidden low‐rank properties in different time windows of the EEG signal and improving the effectiveness of EEG denoising. In the up sampling stage, the first three convolutional layers consist of $\left[\text{TConv}+\text{Conv}+\mathrm{BN}+\text{ReLU}\right]$, where TConv denotes transposed convolution, which serves to gradually restore the scale of the EEG signal and perform feature fusion. The last convolutional layer is composed of $\left[\text{Conv}+\mathrm{BN}+\text{ReLU}\right]$.

#### 3.3.2 Noisy Estimation Module (Net‐N)

This model takes $R^{k-1}$, $N^{k-1}$, and $D^{k-1}$ as inputs, as shown in Figure 2. With δ set to 0.5, $N^{k}$ can be calculated using the following formula:

(16) $$\begin{array}{c} N^{k}=R^{k-1}+N^{k-1}-D^{k-1}-\rho \nabla S\left(N^{k-1}\right) \end{array}$$

For ρ, it is a learnable scalar that is independent of each reconstruction stage. Regarding the Lipschitz continuous gradient function ∇S, studies have proven that a simple CNN composed of convolutional layers and ReLU activation functions satisfies Lipschitz continuity, as do stacks of multiple such simple CNNs [39]. Therefore, based on the principle of simplicity and to avoid complex network designs, we use stacks of simple convolutional layers with ReLU activation functions to approximate ∇S. The network structure is shown in Figure 3B, and we also adopt an effective residual structure to enhance the noise signal for more effective extraction. An updated formula, incorporating the residual enhancement for noise estimation, is given by:

(17) $$\begin{array}{c} N^{k}=R^{k-1}+N^{k-1}-D^{k-1}-\rho \nabla S\left(N^{k-1}+R^{k-1}-D^{k-1}\right) \end{array}$$

#### 3.3.3 Raw EEG Synthesis Model (Net‐R)

To maintain consistency with the iterative process of LRR, we design Net‐R to synthesize new raw input signals *R* from the extracted clean EEG signals and noise.

(18) $$\begin{array}{c} R^{k}=\text{NetR}\left(N^{k}+D^{k}\right) \end{array}$$

Instead of using residual blocks or other complex networks in Net‐R, we utilize a simple and efficient CNN to learn the characteristics of raw EEG signals [40] and effectively synthesize new raw input signals from clean EEG signals and noise. The specific structure is shown in Figure 3C.

In summary, based on the LRR theory and with the aid of deep unfolding techniques, we unfold its iterative process into the designed neural network. For the extraction of clean EEG signals, we cleverly combine prior information in the time‐domain windows of EEG signals with the advantages of Unet, leveraging the potential low‐rank property of one‐dimensional EEG signals to accurately recover clean EEG signals from noisy ones. For the extraction of noise signals and the synthesis of raw EEG signals, we design simple CNN networks based on the principle of simplicity, achieving good results despite the simplicity of the network models.

## 4 Network Training

In this chapter, we first introduce the widely used labeled EEG denoising datasets, namely EEGDenoiseNet and semi‐simulated EEG/EOG, as well as the four‐class classification dataset provided by the MNE software package, known as the MNE M/EEG dataset. We then delve into the preprocessing procedure for the EEGDenoiseNet dataset, aiming to make it more effective for training the proposed LRR‐Unet model. Following that, we present the specific implementation details of the training process.

### 4.1 EEGDenoiseNet

To demonstrate the effectiveness of the LRR‐Unet model for EEG signal denoising, this paper employs a widely adopted EEG signal dataset [41]. Specifically, the dataset contains 4514 clean EEG segments as ground truth, 3400 pure electrooculogram (EOG) segments, and 5598 pure electromyogram (EMG) segments, representing ocular artifacts and myogenic artifacts, respectively. Each segment has a sampling duration of 2 s and a sampling rate of 256 Hz, resulting in signal segments of length 512. Simulated noisy EEG signals can be generated according to Equation (19), where x represents the clean EEG signal, *N* represents either the EOG or EMG noise signal, and the signal‐to‐noise ratio (SNR) of the noisy EEG signal y can be adjusted within the range of −7 dB to 2 dB by tuning the value of λ, thereby enabling EEG signal denoising under different noise intensities.

(19) $$\begin{array}{c} y=x+\mathrm{\lambda N} \end{array}$$

The specific preprocessing steps for the dataset are as follows:

1. First, the EEG signals are randomly shuffled *n* times (in this case, 10 times due to 10 initial SNR levels), and the shuffled results are concatenated together.
2. For the training set, assuming its dimension is (num, feature_num), num random numbers between −7 dB and 2 dB are generated. For each EEG signal, the value of *λ* is calculated based on its corresponding random number, and then the noisy EEG signal *y* is obtained using the above formula.
3. For the test set and validation set, 10 deterministic numbers between −7 and 2 are generated. For each EEG signal, *λ* is calculated based on its corresponding number, and then the noisy EEG signal is obtained using the above formula.
4. The processed EEG signals are standardized.

By following these preprocessing steps, the dataset is prepared in a format suitable for training and evaluating the LRR‐Unet model, enabling it to effectively learn to denoise EEG signals under various noise conditions.

### 4.2 Semi‐Simulated EEG/EOG [42]

The dataset is utilized to evaluate the performance of the proposed model in removing artifacts from multichannel EEG signals. This dataset comprises 19‐channel EEG signals and corresponding EOG signals from 27 subjects. The signals are sampled at a rate of 200 Hz, and all signals are segmented into one‐dimensional segments of 30 s each. The EEG signals undergo band‐pass filtering between 0.5 and 40 Hz and notch filtering at 50 Hz, while the EOG signals undergo band‐pass filtering between 0.5 and 5 Hz. The dataset includes “Contaminated_EEG,” which represents the EEG signals artificially contaminated with artifacts, and “Pure_EEG,” which represents the clean EEG signals collected during eye closure.

### 4.3 CHB‐MIT Dataset

We utilized a real‐world EEG signal dataset to verify the feasibility of the model in practical applications, which was selected from the Children's Hospital Boston (CHB)–Massachusetts Institute of Technology (MIT) scalp EEG database [43]. The database collected EEG recordings of epileptic patients according to the international 10–20 system. The sampling rate of the signal is 256 Hz. The database can be obtained from the PhysioNet website: http://physionet.org/physiobank/database/chbmit/.

### 4.4 MNE M/EEG Dataset

The MNE software package provides a sample dataset that includes combined recordings of MEG, EEG, and EOG from a single participant, with EEG signals collected simultaneously through 60 electrodes. This dataset is a four‐class classification dataset that includes auditory stimuli (delivered monaurally to the left or right ear) and visual stimuli (displayed in the left or right visual hemisphere). In this paper, we utilize this dataset to validate the benefits of our method in practical EEG tasks.

### 4.5 Implementation Details

We utilized the deep learning framework PyTorch to construct our LRR‐Unet model. A simple and efficient Mean Squared Error Loss (MSELoss) was adopted as the loss function. The Adam optimizer was chosen as the optimization algorithm, with a learning rate set to 1e‐4. To prevent overfitting, an early‐stopping strategy was implemented.

## 5 Experiment

In this chapter, we first introduce three advanced algorithms for EEG signal denoising. Then, we present the evaluation metrics used for both denoising and classification tasks. Through ablation experiments, we validate the effectiveness of our proposed model and study the impact of some key hyperparameters on its performance. Finally, we conduct both qualitative and quantitative comparisons with some state‐of‐the‐art EEG denoising models to verify the performance of our method.

### 5.1 Comparing Methods

*1D‐ResCNN* [22]: This network primarily consists of three parallel submodules with different convolutional kernels and residual connections. Each one‐dimensional convolutional layer is followed by a batch normalization layer and a ReLU activation function.

*DeepSeparator* [23]: DeepSeparator is an end‐to‐end deep learning framework that does not rely on human‐designed prior assumptions and knowledge about artifacts. It can be viewed as a nonlinear decomposition and reconstruction of the input signal, extending linear blind source separation methods. The encoder captures and amplifies features in the original electroencephalogram (EEG), the separator extracts trends, detects, and suppresses artifacts in the embedding space, and the decoder reconstructs the EEG signal and artifacts.

*EEGDnet* [24]: This model incorporates a Transformer architecture, which consists of four components: a reshape layer, self‐attention blocks, feedforward blocks, and normalization layers. Specifically, the one‐dimensional EEG signal is first reshaped into a two‐dimensional form and then fed into a two‐dimensional Transformer encoder. The encoder is composed of alternating self‐attention blocks and feedforward blocks. Normalization layers and residual connections are applied after each block. The output of the Transformer encoder is reshaped back into one dimension as the final output.

*IC‐U‐Net*: Based on the U‐Net architecture and a collection of loss functions, IC‐U‐Net employs synthetically mixed brain and nonbrain independent components (ICs) separated by ICA and ICLabel. During the training process, it identifies model parameters that minimize the difference between the mixed brain ICs and the model's outputs.

### 5.2 Efficiency of Artifacts Removal

We use four metrics to evaluate the denoising results: Relative Root Mean Square Error in the time domain (RRMSE_temporal), Relative Root Mean Square Error in the spectral domain (RRMSE_spectral), Correlation Coefficient (CC), and Signal‐to‐Noise Ratio (SNR). Firstly, considering the importance of time‐domain information in EEG signals, we use RRMSE_temporal to quantify the relative error between the denoised signal and the original signal in the time domain. Secondly, due to the rich spectral information contained in EEG, we employ RRMSE_spectral to quantify the relative error between the denoised signal and the original signal in the frequency domain. CC reflects the linear relationship between the denoised signal and the original signal, which is crucial for maintaining important information about inter‐regional correlations. SNR, a classic evaluation metric in the field of denoising, measures the ratio of the power of the useful signal to the power of the noise signal. The combined use of these metrics helps to comprehensively assess denoising performance in both the time and frequency domains.

The mathematical expressions for RRMSE_temporal, RRMSE_spectral, CC, and SNR are given by Equations (20–23) respectively, where $\mathrm{RMS}\left(\cdot \right)$ denotes the Root Mean Square, $\mathrm{PSD}\left(\cdot \right)$ denotes the Power Spectral Density, $\mathrm{Cov}\left(\cdot \right)$ and $\mathrm{Var}\left(\cdot \right)$ represent covariance and variance respectively, and $P_{s}$ and $P_{n}$ are the effective powers of the signal and noise signal respectively.

(20) $$\begin{array}{c} {\text{RRMSE}}_{\text{temporal}}=\frac{\mathrm{RMS}\left(F\left(y\right)-x\right)}{\mathrm{RMS}\left(x\right)}=\frac{\mathrm{RMS}\left(\hat{x}-x\right)}{\mathrm{RMS}\left(x\right)} \end{array}$$

(21) $$\begin{array}{c} {\text{RRMSE}}_{\text{spectral}}=\frac{\mathrm{RMS}\left(\mathrm{PSD}\left(F\left(y\right)\right)-\mathrm{PSD}\left(x\right)\right)}{\mathrm{RMS}\left(\mathrm{PSD}\left(x\right)\right)}=\frac{\mathrm{RMS}\left(\mathrm{PSD}\left(\hat{x}\right)-\mathrm{PSD}\left(x\right)\right)}{\mathrm{RMS}\left(\mathrm{PSD}\left(x\right)\right)} \end{array}$$

(22) $$\begin{array}{c} \mathrm{CC}=\frac{\mathrm{Cov}\left(F\left(y\right),x\right)}{\sqrt{\mathrm{Var}\left(F\left(y\right)\right)\mathrm{Var}\left(x\right)}}=\frac{\mathrm{Cov}\left(\hat{x},x\right)}{\sqrt{\mathrm{Var}\left(\hat{x}\right)\mathrm{Var}\left(x\right)}} \end{array}$$

(23) $$\begin{array}{c} \mathrm{SNR}=10\mathrm{lg}\frac{P_{s}}{P_{n}} \end{array}$$

### 5.3 Application in Downstream BCI Classification Task

To demonstrate the feasibility of LRR‐Unet in practical applications, we further evaluated it on a BCI classification task. In this study, various artifact removal methods were used to preprocess the data, and then the processed data were used to classify event‐related potentials. We employed accuracy, recall, F1 score, Matthews Correlation Coefficient (MCC), and Kappa statistic to evaluate classification performance. The calculation formulas for each evaluation metric are as follows:

(24) $$\begin{array}{c} \text{Accuracy}=\frac{\mathrm{TP}+\mathrm{TN}}{\mathrm{TP}+\mathrm{FP}+\mathrm{TN}+\mathrm{FN}\;} \end{array}$$

(25) $$\begin{array}{c} \text{Recall}=\frac{\;\mathrm{TP}}{\mathrm{TP}+\mathrm{FN}} \end{array}$$

(26) $$\begin{array}{c} \text{Precision}=\frac{\;\mathrm{TP}}{\mathrm{TP}+\mathrm{FP}} \end{array}$$

(27) $$\begin{array}{c} F1=\frac{2\times \text{Recall}\times P\;\text{recision}}{P\;\text{recision}+\text{Recall}} \end{array}$$

(28) $$\begin{array}{c} \mathrm{MCC}=\frac{T\;P\times T\;N-F\;P\times F\;N}{\sqrt{\left(T\;P+F\;P\;\right)\left(T\;P+F\;N\right)\left(T\;N+F\;P\;\right)\left(T\;N+F\;N\right)}} \end{array}$$

(29) $$\begin{array}{c} \text{Kappa}=\frac{\;2\times \left(\mathrm{TP}\times \mathrm{TN}-\mathrm{FN}\times \mathrm{FP}\;\right)}{\left(\mathrm{TP}+\mathrm{FP}\;\right)\times \left(\mathrm{FP}+\mathrm{TN}\right)+\left(\mathrm{TP}+\mathrm{FN}\right)\times \left(\mathrm{FN}+\mathrm{TN}\right)} \end{array}$$

### 5.4 Ablation Study

#### 5.4.1 Enhancement of Model Performance by Unet

In this paper, based on prior information in one‐dimensional EEG signals, a Unet model is designed to extract clean EEG signals from noisy ones. To verify its effectiveness, we also designed a simple CNN structure for the same purpose and compared the denoising effects of both. The results of various evaluation metrics are presented in the table below.

The results in Table 1 demonstrate that the proposed Unet architecture achieves significantly superior denoising performance compared to conventional CNN structures. This enhancement primarily stems from its ability to (1) capture multi‐scale EEG features during the encoding (down sampling) phase, which facilitates the learning of low‐rank properties across temporal windows, and (2) reconstruct detailed information through skip connections that effectively integrate low‐level and high‐level features during the decoding (up sampling) phase.

**TABLE 1:** Improvement in model performance by Unet.

Method	RRMSE‐t	RRMSE‐s	CC	SNR
LRRnet	0.520	0.551	0.858	6.078
LRR‐Unet	**0.398**	**0.459**	**0.918**	**8.206**

*Note:* Significance: The bold values represent the optimal results. Analysis reveals that the Unet architecture proposed in this paper outperforms the traditional CNN model across all evaluation metrics, which proves the effectiveness of Unet.

#### 5.4.2 Impact of Iteration Count K on Model Performance

To identify the optimal number of iterations, we conducted experiments using different iteration counts (the number of stacked layers in the entire model). The experimental results are presented in the table below:

Based on the Table 2, it can be concluded that the model achieves better performance when *K* equals 2 or 3. Specifically, when *K* equals 2, the model demonstrates superior removal of EMG noise. When *K* equals 3, the model exhibits better denoising of EOG signals. However, the denoising effect of the model does not improve as the iteration count *K* increases beyond this point. Taking all factors into consideration, we have chosen *K* equals 3 for all subsequent experiments.

**TABLE 2:** Impact of iteration count *K* on model performance.

Contaminated by EOG				Contaminated by EMG				
Stages (*K*)	RRMSE‐t	RRMSE‐s	CC	SNR	RRMSE‐t	RRMSE‐s	CC	SNR
1	0.533	0.573	0.869	5.717	0.677	0.689	0.797	3.702
2	0.426	0.477	0.911	7.571	**0.547**	**0.503**	**0.831**	**5.527**
3	**0.398**	0.459	**0.918**	**8.206**	0.549	0.529	**0.831**	5.449
4	0.413	0.450	0.911	7.857	0.570	0.537	0.820	5.085
5	0.413	**0.447**	0.912	7.875	0.554	0.517	0.829	5.376
6	0.421	0.453	0.908	7.676	0.564	0.527	0.823	5.214
7	0.487	0.499	0.877	6.341	0.634	0.575	0.779	4.107

*Note:* Significance:The bold values represent the optimal results among all evaluation metrics. The model achieved relatively good performance when K was set to 2 or 3; after comprehensive consideration, K = 3 was selected as the final parameter setting.

### 5.5 Performance Comparison of Different Algorithms

#### 5.5.1 Model Deployment I via EEGDenoiseNet Dataset

To demonstrate the effectiveness of the proposed model in this paper, we compared the proposed LRR‐Unet model with four existing deep learning‐based EEG denoising methods. Tables 3 and 4 show the denoising effects of various methods on EOG and EMG datasets.

**TABLE 3:** Average performance of each method after denoising under initial signal‐to‐noise ratios of −7 to 2 dB for EEG + EOG.

Method	RRMSE‐t	RRMSE‐s	CC	SNR
LRR‐Unet	**0.398**	**0.459**	**0.918**	**8.206**
DeepSeparator	0.482	0.528	0.885	6.465
1D‐ResCNN	0.495	0.454	0.867	6.229
EEGDnet	0.522	0.479	0.853	5.732
IC‐U‐Net	0.475	0.463	0.881	6.563

*Note:* Significance: The bold values represent the optimal results. Analysis indicates that for EOG noise removal, the model proposed in this paper achieved the best performance in terms of RRMSE‐t, RRMSE‐s, CC, and SNR. This proves that the proposed model is significantly superior to other algorithms across multiple dimensions.

**TABLE 4:** Average performance of each method after denoising under initial signal‐to‐noise ratios of −7 to 2 dB for EEG + EMG.

Method	RRMSE‐t	RRMSE‐s	CC	SNR
LRR‐Unet	**0.549**	**0.529**	**0.831**	**5.449**
DeepSeparator	0.575	0.544	0.819	5.031
1D‐ResCNN	0.627	0.561	0.781	4.159
EEGDnet	0.605	0.542	0.798	4.496
IC‐U‐Net	0.577	0.546	0.819	4.961

*Note:* Significance:The bold values represent the optimal results. Analysis indicates that for EMG noise removal, the model proposed in this paper achieved the best performance in terms of RRMSE‐t, RRMSE‐s, CC, and SNR. This proves that the proposed model is significantly superior to other algorithms across multiple dimensions.

**FIGURE 4:**
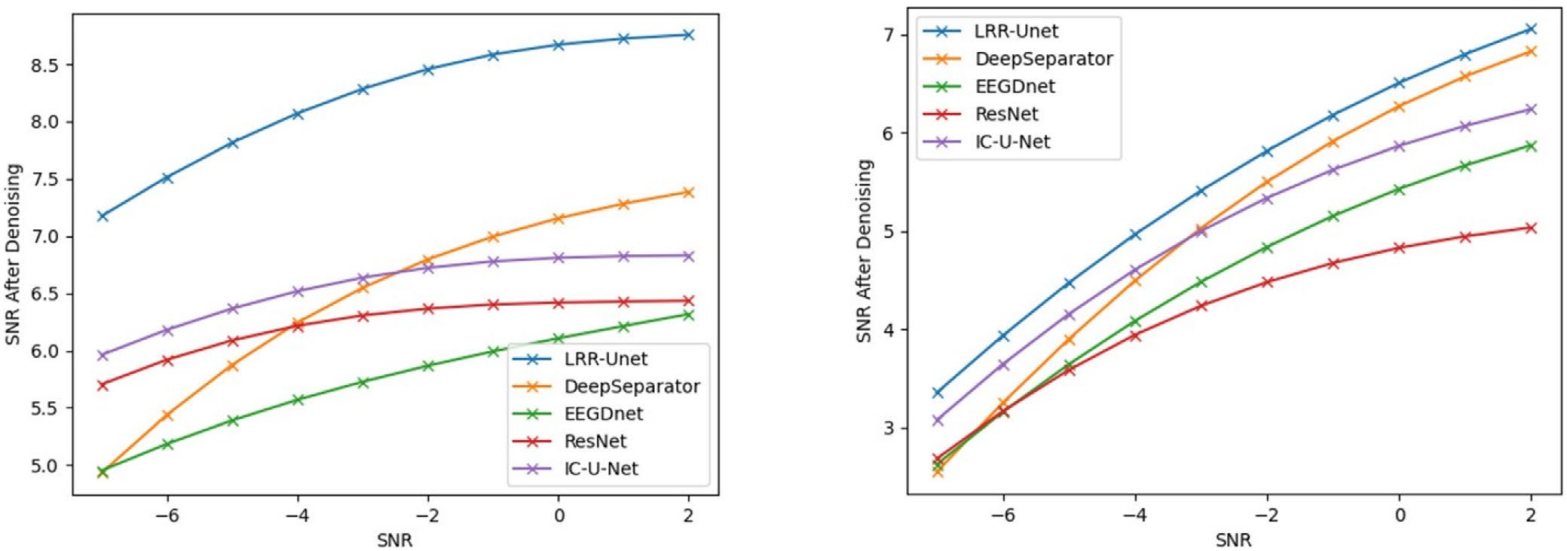
Comparison of the signal‐to‐noise ratio (SNR) after denoising electroencephalograms (EEGs) contaminated by electrooculograms (EOGs) on the left and electromyograms (EMGs) on the right using various algorithms under the initial signal‐to‐noise ratios ranging from −7 to 2 decibels (dB). Here, the vertical axis represents the signal‐to‐noise ratio, and the horizontal axis represents the initial signal‐to‐noise ratio.

**FIGURE 5:**
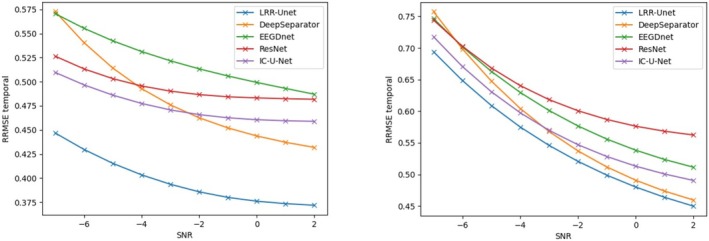
Comparison of the relative root mean square error in the time domain (RRMSE‐t) after denoising electroencephalograms (EEGs) contaminated by electrooculograms (EOGs) (left) and electromyograms (EMGs) (right) using various algorithms under initial signal‐to‐noise ratios ranging from −7 dB to 2 dB. The vertical axis represents the root mean square error in the time domain, and the horizontal axis represents the initial signal‐to‐noise ratio.

**FIGURE 6:**
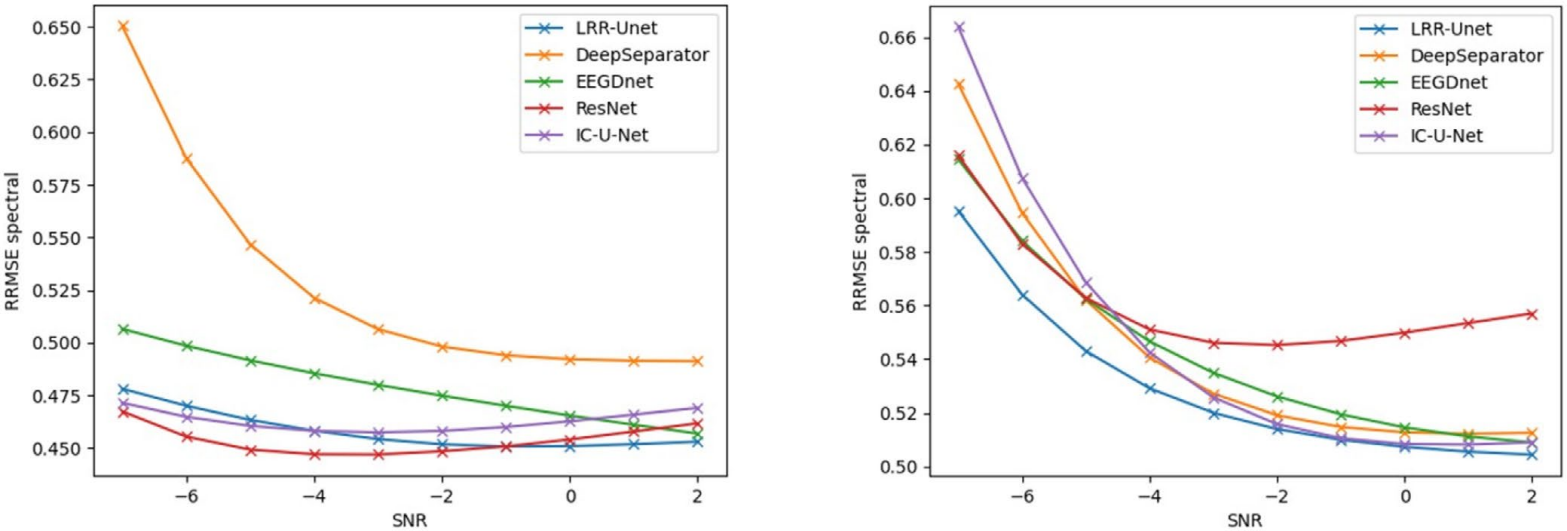
Comparison of the relative root mean square error in the spectral domain (RRMSE‐s) after denoising electroencephalograms (EEGs) contaminated by electrooculograms (EOGs) (left) and electromyograms (EMGs) (right) using various algorithms under initial signal‐to‐noise ratios ranging from −7 dB to 2 dB. The vertical axis represents the root mean square error in the spectral domain, and the horizontal axis represents the initial signal‐to‐noise ratio.

**FIGURE 7:**
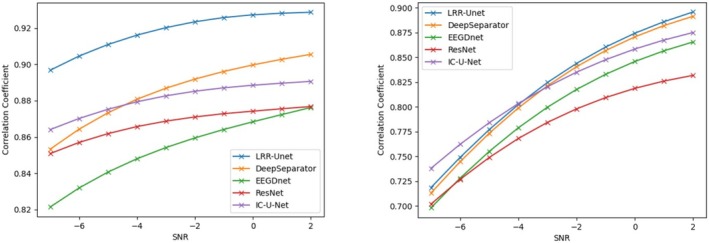
Comparison of the correlation coefficient (CC) after denoising electroencephalograms (EEGs) contaminated by electrooculograms (EOGs) on the left and electromyograms (EMGs) on the right using various algorithms under the initial signal‐to‐noise ratios ranging from −7 to 2 decibels (dB). Here, the vertical axis represents the difference in the correlation coefficient, and the horizontal axis represents the initial signal‐to‐noise ratio.

This study uses two key properties: (1) the similarity of EEG signals across time windows, and (2) the concentrated energy of EOG and EMG noise in specific time or frequency ranges. We combine these with LRR theory's low‐rank properties using a deep unfolding network with Unet architecture. Results show our model outperforms others in removing both EOG and EMG artifacts (see tables). This advantage comes from effectively using prior knowledge about EEG signals and noise patterns, combined with data‐driven learning. Most traditional deep learning methods ignore such prior knowledge, limiting their performance. Figures 4, 5, 6, 7 compare denoising results across different initial SNR levels. Our model maintains better performance than others at all noise levels, proving its robustness.

**FIGURE 8:**
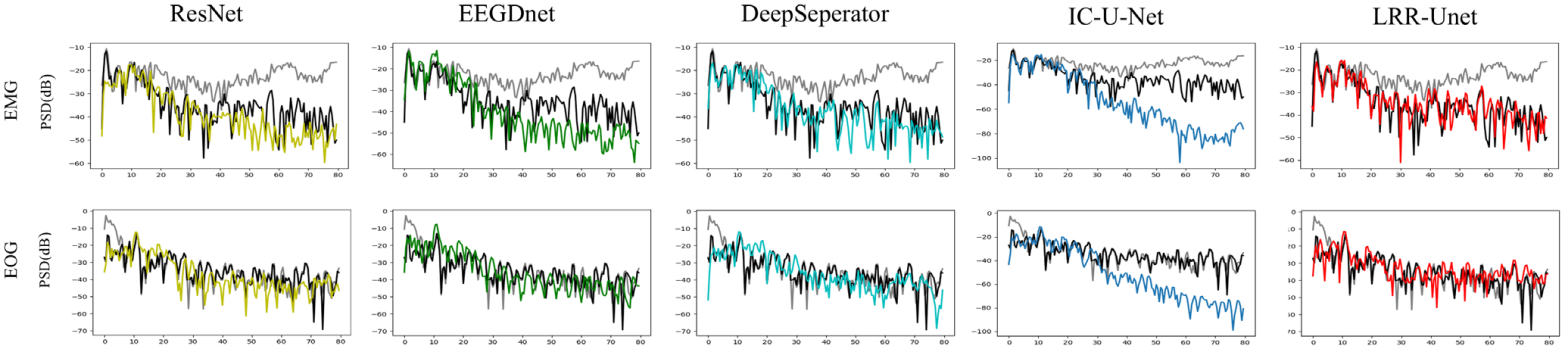
In the EEGDenoiseNet Dataset, the PSD (power spectral density) graph of the denoised EEG signals by the algorithm proposed in this paper and other algorithms is presented. The light gray curves represent the original EEG signals with different artifacts, the black line represents the true clean EEG signal, and the other colors represent the denoised EEG signals.

From the perspective of the spectral analysis results shown in Figure 8, the PSD (power spectral density) lines of the algorithm proposed in this paper exhibit the highest degree of fitting with the black line among those affected by both EMG and EOG noise (the light gray lines represent the original EEG signals with different artifacts, while the black line represents the true clean EEG signal). This indicates that the EEG signals reconstructed by the algorithm in this paper have effectively removed noise components and successfully extracted useful frequency information. In contrast, other deep learning models fail to adequately incorporate prior knowledge of EEG signals and noise characteristics, resulting in the loss of valid neural information during noise removal and consequently compromising denoising performance.

**FIGURE 9:**
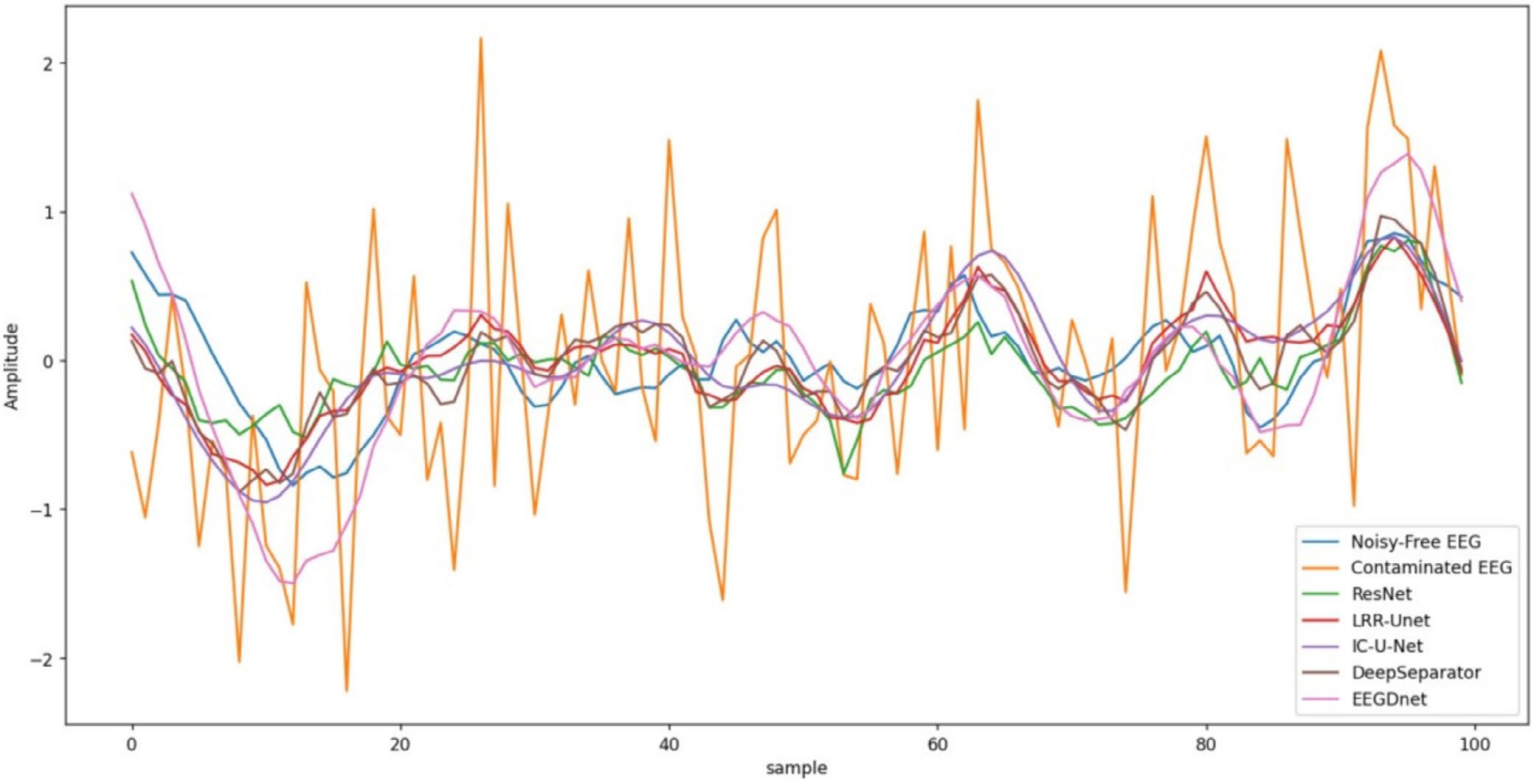
In the EEGDenoiseNet Dataset, the visualization of the denoising effects of various algorithms on electroencephalograms (EEGs) contaminated by electrooculograms (EMG).

**FIGURE 10:**
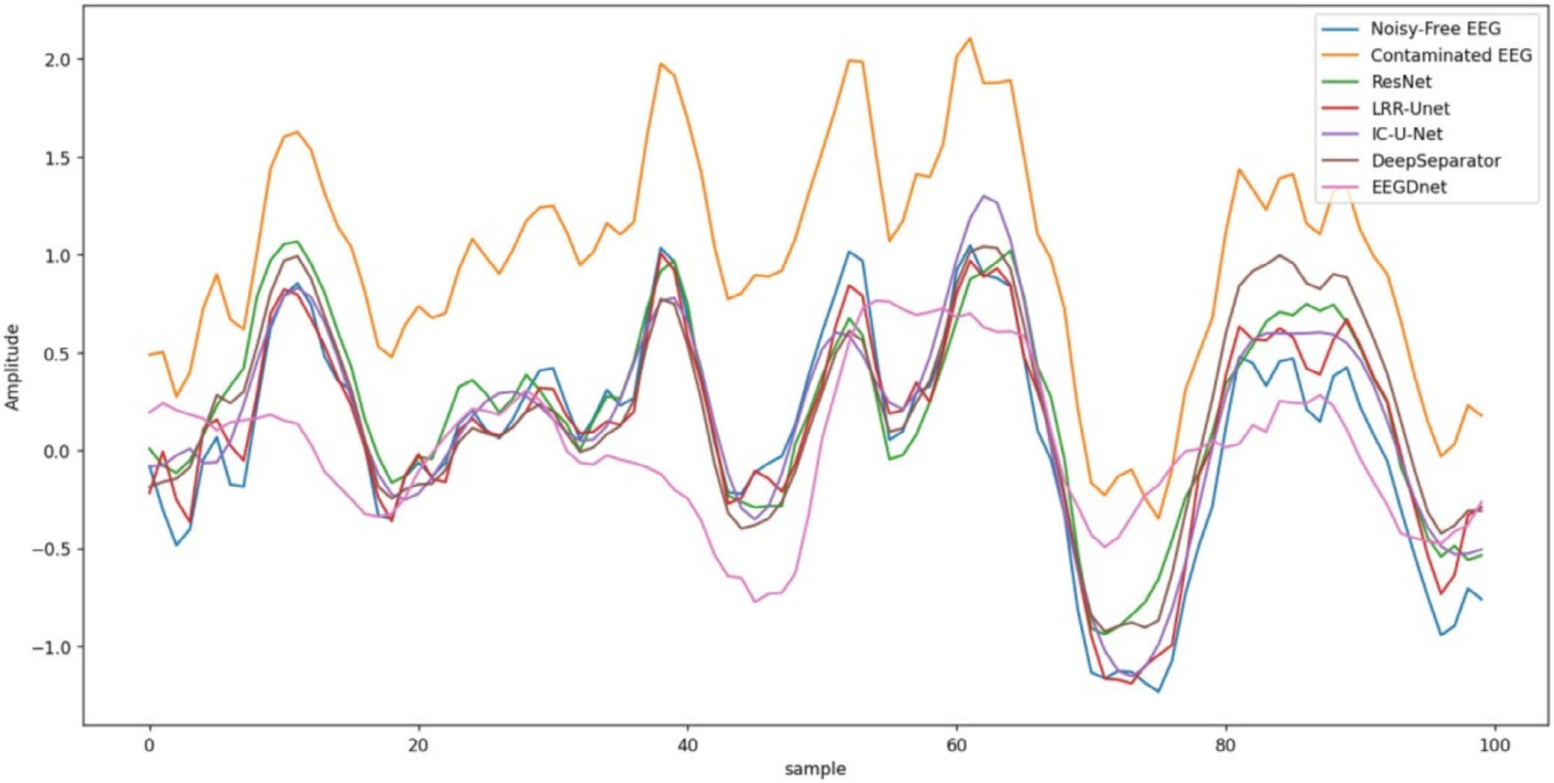
In the EEGDenoiseNet Dataset, the visualization of the denoising effects of various algorithms on electroencephalograms (EEGs) contaminated by electrooculograms (EOG).

Figures 9 and 10 present partial visualization results of different algorithms for EOG and EMG artifact removal. While all models demonstrate certain denoising effects on contaminated EEG signals, qualitative evaluations confirm that our proposed model achieves superior performance in both global trend tracking and local detail preservation compared to other approaches. The denoised signals exhibit closer approximation to clean EEG recordings, which can be attributed to our model's comprehensive consideration of the inherent differences between neural signals and artifacts. Specifically, we developed a dedicated EEG denoising framework that rigorously integrates the denoising process with low‐rank recovery theory. This methodology effectively preserves critical neural characteristics in the denoised output—a crucial advantage for subsequent EEG analysis and interpretation.

#### 5.5.2 Model Deployment II via Semi‐Simulated EEG/EOG

**FIGURE 11:**
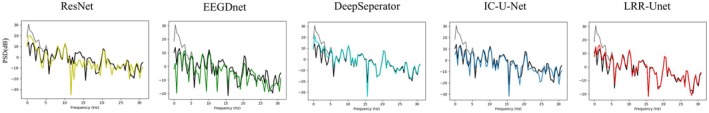
Example of PSD (power spectral density) results after removing eye artifacts from multi‐channel EEG signals.

**FIGURE 12:**
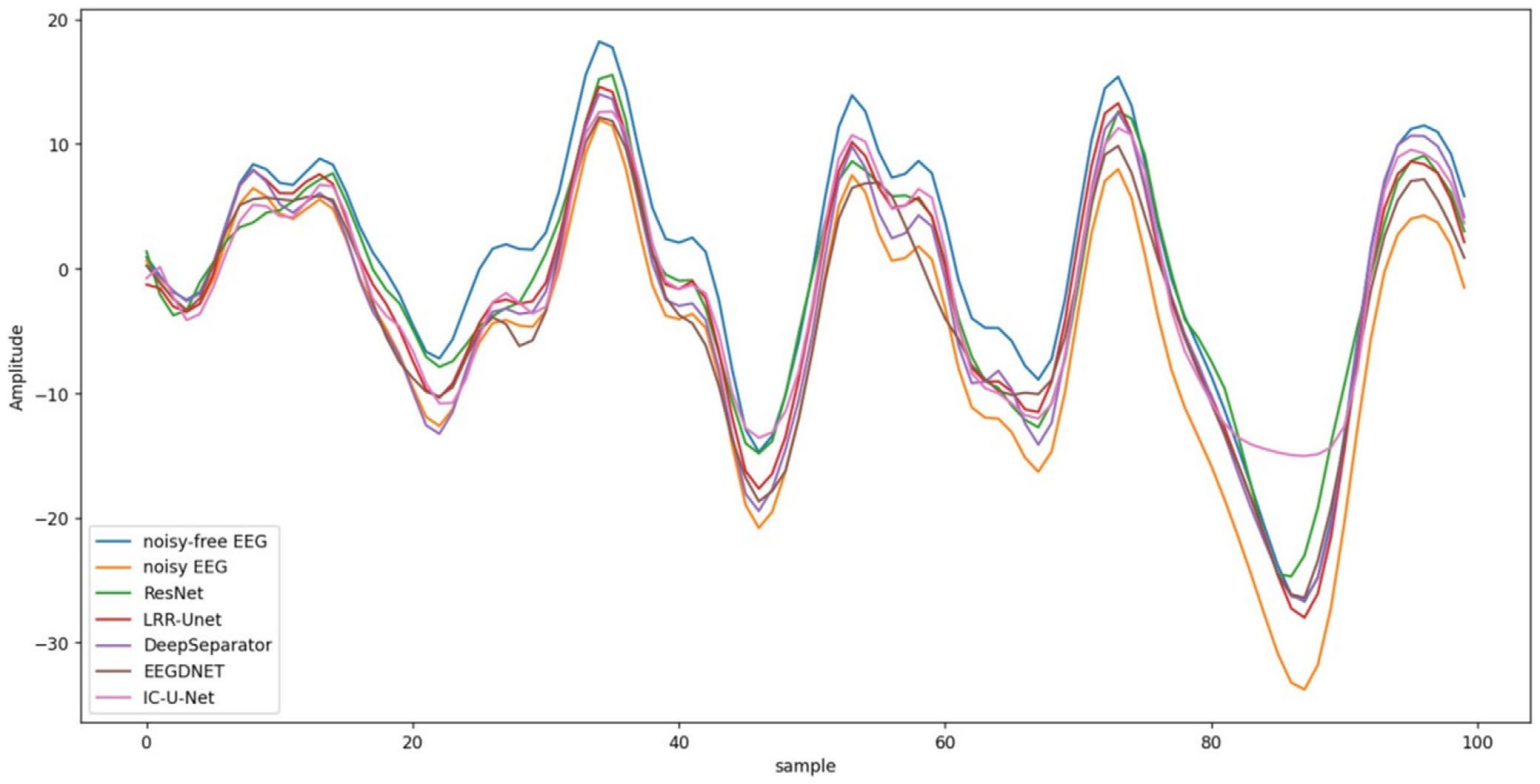
Visual representation of denoising effects of various algorithms on EEG signals contaminated by eye artifacts from multi‐channel EEG signals.

To validate the denoising performance of our model for multi‐channel EEG signals, we conducted extensive experiments on the “semi‐simulatedEEG/EOG” dataset. Quantitative evaluation metrics presented in Table 5 demonstrate that our algorithm achieves optimal performance across all assessment criteria. Furthermore, experiments conducted on diverse datasets confirm the model's superior cross‐dataset generalization capability. From the perspective of the spectral analysis results shown in Figure 11, our algorithm's power spectral density (PSD) curves show the highest correlation with the reference clean EEG signals (black line) under both EMG and EOG contamination (light gray lines represent original artifact‐laden EEG signals). This indicates that our reconstructed EEG signals effectively eliminate noise components while accurately preserving useful frequency information. Figure 12 presents comparative visualization results of multi‐channel EEG signals contaminated by EOG artifacts after denoising. Qualitative analysis reveals that our model's output most closely approximates the clean EEG reference, particularly in preserving critical signal details—a crucial advantage for subsequent EEG studies. These results further validate our approach of systematically integrating prior knowledge of EEG characteristics and noise properties with data‐driven deep learning.

**TABLE 5:** Performance of various denoising methods on the multi‐channel EEG signal dataset.

Denoising method	RRMSE‐t	RRMSE‐s	CC	SNR
LRR‐Unet	**0.389**	**0.475**	**0.920**	**9.362**
DeepSeparator	0.451	0.610	0.891	8.198
1D‐ResCNN	0.520	0.610	0.859	6.112
EEGDnet	0.479	0.481	0.874	6.845
IC‐U‐Net	0.492	0.597	0.891	6.534

*Note:* Significance: The bold values represent the optimal results. Analysis shows that for multi‐channel EEG signals, the RRMSE‐t, RRMSE‐s, CC, and SNR of the signals denoised by the proposed algorithm are all the best. This proves that the proposed algorithm not only has advantages in single‐channel signal denoising, but also exhibits strong generalization ability in multi‐channel EEG signal denoising, thus demonstrating good practicality.

#### 5.5.3 Model Deployment III via CHB‐MIT

**FIGURE 13:**
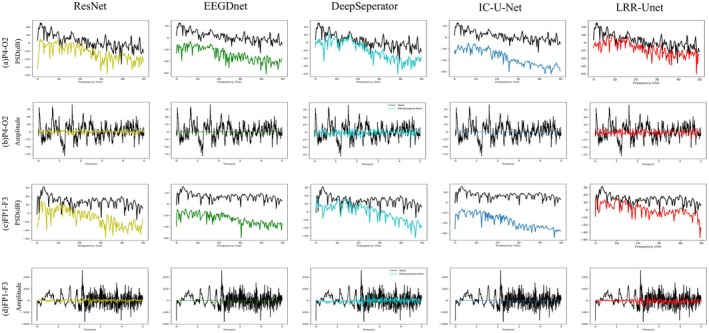
Example of experimental waveform and PSD (power spectral density) results for artifact removal in the real EEG dataset CHB‐MIT.

Figure 13 demonstrates the artifact removal performance of multiple algorithms on two segments of real electroencephalogram (EEG) signals. Figure 13b,d present the raw EEG signals recorded from the P4‐O2 and FP1 ~ F3 channels, respectively. As illustrated, these EEG signals are heavily contaminated by EMG artifacts, EOG artifacts, and transient motion artifacts. A comparative analysis of the EEG reconstruction results between LRR‐Unet and other algorithms reveals that LRR‐Unet effectively suppresses noise while better preserving meaningful neural information. In contrast, conventional methods inevitably introduce signal distortion during noise suppression. For further evaluation, Figure 13a,c display the power spectral density (PSD) of the reconstructed EEG signals, corresponding to the waveforms in Figure 13b,d, respectively. The results indicate that all algorithms successfully attenuate both high‐ and low‐frequency artifact components. However, unlike LRR‐Unet, other approaches lead to excessive attenuation of uncontaminated spectral components, resulting in loss of critical EEG information. This limitation stems from the fact that most traditional deep learning methods neglect prior knowledge of EEG signals and noise, whereas the proposed LRR‐Unet integrates domain‐specific priors with data‐driven deep learning, thereby achieving superior denoising performance without sacrificing signal fidelity. This experiment validates the reliability and practical applicability of the proposed LRR‐Unet model in real‐world scenarios.

#### 5.5.4 Model Deployment IV via BCI Experiment

The elimination of artifacts in electroencephalogram (EEG) data can have a significant impact on subsequent analysis processes, particularly on the performance of Brain–Computer Interfaces (BCI). Therefore, this section focuses on exploring whether EEG denoising techniques can optimize the prediction of Event‐Related Potentials (ERP), which are characteristic neural responses generated by the brain in response to novel stimuli. In this study, we utilized the pyRiemann tool [44], which is based on the principles of Riemannian geometry for symmetric positive‐definite (SPD) matrices, to classify ERP EEG data from a four‐class classification task. Prior to ERP classification, we applied various denoising algorithms used in the literature to preprocess the EEG signals and systematically evaluated the effectiveness of these methods in subsequent analysis tasks. Considering the difficulty in obtaining completely noise‐free EEG training data in practical tasks, we decided to directly apply the denoising models trained on previous datasets. This approach also provided us with an opportunity to test the generalization ability of the models.

This study employed a 10‐fold cross‐validation approach to evaluate the classification performance of various algorithms on preprocessed EEG signals. The normality of all experimental data was first verified using the Shapiro–Wilk test. Subsequently, paired *t*‐tests were conducted to analyze the statistical significance of differences between the proposed LRR‐Unet model and comparative models. The results demonstrated statistically significant differences (*p* < 0.01 for all comparisons). The detailed evaluation metrics (mean ± standard deviation) are presented in Table 6.

**TABLE 6:** Classification performance of BCI data set, the normality of all datasets was confirmed by the Shapiro–Wilk test, with the mean ± standard deviation of each evaluation metric summarized in the table.

	Raw	DeepSeparator	1D‐ResCNN	EEGDnet	IC‐U‐Net	LRR‐Unet
Accuracy	0.8340 ± 0.0494	0.7452 ± 0.0629	0.6818 ± 0.1111	0.7602 ± 0.0829	0.7177 ± 0.0852	**0.8797** ± **0.0427**
Recall	0.8335 ± 0.0621	0.7772 ± 0.0542	0.6904 ± 0.1181	0.7836 ± 0.0758	0.7383 ± 0.1042	**0.8874** ± **0.0489**
Precision	0.8390 ± 0.0663	0.7714 ± 0.0664	0.6786 ± 0.0914	0.7744 ± 0.0710	0.7334 ± 0.0973	**0.8784** ± **0.0461**
F1 Score	0.8193 ± 0.0658	0.7378 ± 0.0564	0.6651 ± 0.1067	0.7508 ± 0.0785	0.7109 ± 0.1004	**0.8708** ± **0.0450**
MCC	0.7771 ± 0.0641	0.6740 ± 0.0904	0.5819 ± 0.1386	0.6909 ± 0.1118	0.6318 ± 0.1138	**0.8388** ± **0.0627**
Kappa	0.7683 ± 0.0664	0.6525 ± 0.0900	0.5693 ± 0.1446	0.6729 ± 0.1181	0.6151 ± 0.1171	**0.8321** ± **0.0631**

*Note:* Significance: The bold values represent the optimal results. The classification performance of EEG signals preprocessed by the proposed algorithm in this paper achieved the best results across all evaluation metrics. Meanwhile, it significantly improved the classification performance compared with the original EEG signals. This proves that the denoising algorithm in this paper can not only remove noise from EEG signals, but also retain the useful information in the original EEG signals to the greatest extent, thus verifying its practical value.

Based on the experimental results, we can conclude that classification results using data preprocessed by our proposed LRR‐Unet demonstrate superior performance in both recall and precision metrics compared to raw signals, indicating effective artifact removal and signal feature enhancement. Notably, our model achieves significantly better classification outcomes than other algorithms, which stem from its systematic incorporation of prior knowledge about both EEG signals and noise characteristics. Specifically, LRR‐Unet rigorously integrates the denoising process with low‐rank recovery theory by fundamentally addressing the inherent distinctions between neural signals and artifacts. In contrast, data denoised by other methods exhibit degraded classification performance relative to original signals. This limitation arises because these models rely solely on data‐driven training without adequately considering the prior knowledge of EEG and noise properties, consequently attenuating useful neural information during artifact removal. The comparative classification results unequivocally demonstrate LRR‐Unet's significant advantages over alternative methods, confirming its unique capability to preserve clinically relevant information while denoising—a critical advancement for EEG analysis and research.

## 6 Conclusion

In this paper, we delve deeply into EEG denoising methods based on low‐rank recovery theory and deep unfolding networks, proposing an innovative solution aimed at efficiently extracting pure neural activity information from noise‐contaminated electroencephalogram (EEG) signals. This method integrates low‐rank recovery techniques with deep unfolding networks, using a certain number of neural network layers to simulate the iterative optimization process of low‐rank recovery techniques, thereby addressing the poor interpretability of deep learning algorithms. It ingeniously leverages the Unet to capture the underlying low‐rank structure in noisy EEG signals. Experimental results show that our method demonstrates significant denoising performance on multiple EEG datasets, not only outperforming existing methods in removing different types of noise (such as eye movement artifacts, electromyographic interference, etc.), but also excelling in preserving the physiological characteristics of EEG signals. Additionally, we conducted brain–computer interface (BCI) application experiments, which showed higher classification accuracy when using our algorithm for EEG signal preprocessing, thereby validating the robustness, generalization, and potential application prospects of our LRR‐Unet method in the fields of neuroscience and neuro engineering based on EEG signals. At the theoretical level, this paper introduces in detail how to perform theoretical derivations based on the low‐rank and sparse components of low‐rank recovery theory, and then use appropriate neural networks for computation, gradually approaching the true low‐rank solution during the iterative process and learning key features in EEG signals. These findings not only deepen our understanding of the EEG denoising process but also provide useful insights for the improvement of future algorithms.

Despite preliminary results, this study still has some limitations. Currently, the method primarily focuses on denoising EEG signals in the time domain and has not fully considered information in the frequency domain. Therefore, extending low‐rank recovery theory and deep unfolding networks to the time‐frequency domain will be an important direction for future research.

In summary, the EEG denoising method proposed in this study, based on low‐rank recovery theory and deep unfolding networks, provides an effective preprocessing tool for the field of EEG signal processing and is expected to promote the in‐depth development of neuroscience and neuroengineering research.

## Conflicts of Interest

The authors declare no conflicts of interest.

## Data Availability

The data that support the findings of this study are available in public repositories via the following Uniform Resource Identifiers (URIs): EEGDenoiseNet via GitHub URI (https://github.com/ncclabsustech/EEGdenoiseNet), the Semi‐Simulated EEG/EOG dataset via Mendeley Data URI (https://data.mendeley.com/datasets/wb6yvr725d/1), the CHB‐MIT Dataset via PhysioNet URI (http://physionet.org/physiobank/database/chbmit/), and the MNE M/EEG Dataset via the MNE‐Python Documentation Repository URI (https://mne.tools/stable/api/datasets.html#module‐mne.datasets).
